# Seasonal variation in cuckoldry rates in the socially monogamous cichlid fish *Variabilichromis moorii*

**DOI:** 10.1007/s10750-022-05042-0

**Published:** 2022-10-18

**Authors:** Holger Zimmermann, Aneesh P. H. Bose, Helgit Eisner, Jonathan M. Henshaw, Angelika Ziegelbecker, Florian Richter, Sandra Bračun, Cyprian Katongo, Karoline Fritzsche, Kristina M. Sefc

**Affiliations:** 1 Institute of Biology, University of Graz, Universitätsplatz 2, 8010 Graz, Austria; 2 Department of Biological Sciences, University of Zambia, Great East Road Campus, P.O. Box 32379, Lusaka, Zambia; 3 Present Address: Institute of Vertebrate Biology, Czech Academy of Sciences, Brno, Czech Republic; 4 Present Address: Department of Wildlife, Fish and Environmental Studies, Swedish University of Agricultural Sciences (SLU), 90183 Umeå, Sweden; 5 Present Address: Institute of Molecular Biosciences, University of Graz, Graz, Austria; 6 Present Address: Institute of Biology I, University of Freiburg, Hauptstraße 1, 79104 Freiburg, Germany

**Keywords:** Extra-pair paternity, Cuckoldry, Lake Tanganyika, Parentage analysis, Mating system, Seasonal variation

## Abstract

Mating patterns in animal populations can respond to environmental conditions and consequently vary across time. To examine this variation in nature, studies must include temporal replicates from the same population. Here, we report temporal variation in genetic parentage in the socially monogamous cichlid *Variabilichromis moorii* from Lake Tanganyika, using samples of broods and their brood-tending parents that were collected across five field trips from the same study population. The sampled broods were either spawned during the dry season (three field trips) or during the rainy season (two trips). In all seasons, we detected substantial rates of extra-pair paternity, which were ascribed to cuckoldry by bachelor males. Paternity shares of brood-tending males were consistently higher, and the numbers of sires per brood were consistently lower, in broods that were spawned in the dry seasons compared to broods from the rainy seasons. In contrast, the strength of size-assortative pairing in our *V. moorii* population did not vary temporally. Seasonal fluctuations in environmental conditions, such as water turbidity, are proposed as a mechanism behind variable cuckolder pressure. Our data demonstrate the utility of long-term monitoring to improve our understanding of animal mating patterns.

**Supplementary Information:**

The online version contains supplementary material available at 10.1007/s10750-022-05042-0.

## Introduction

Animal-mating patterns have profound effects on evolutionary processes. Mating patterns determine how an important resource—mating opportunities—is distributed among individuals in a population, often with unequal distributions between the sexes. For instance, if females are unavailable for mating for prolonged periods of time due to gestation or brood care, then it is generally expected that males will compete more intensely among one another for the few available females. The resulting competition for mates can give rise to sexual selection on traits that are positively associated with mating success, sometimes leading to the evolution of exaggerated ornaments or armaments (Andersson, 2019). How mating opportunities are distributed among individuals can also influence their decisions regarding how to allocate their time and energy, for instance, when choosing between the pursuit of new reproduction or investing into care for current offspring (Fromhage & Jennions, 2016).

The expectation that mating success is positively related to reproductive success (i.e. the number of progeny produced) led to the assumption that variance in reproductive success can be roughly predicted from the social mating system. For example, social monogamy is considered to be associated with mild variance in the mating success of both sexes, since each reproductive adult will claim only one member of the opposite sex from the mating pool (Avise et al., 2002). In contrast, mating success can become more variable and skewed as more individuals engage in polygamous matings, which can leave fewer mating opportunities for less successful consexuals. Extreme examples are found, for example, in pinniped leks, where a minority of males monopolizes the vast majority of copulations (Fabiani et al., 2004). Consequently, social mating system classifications are often used as convenient proxies for the intensity of sexual selection in evolutionary studies (e.g. Gonzalez-Voyer et al., 2008).

However, the levels of reproductive success variance that occur across individuals may sometimes be difficult to accurately estimate based on inspections of the social mating system alone. For instance, brood parentage may differ substantially from expectations based on the social mating system. This has been famously demonstrated in birds, where the majority of socially monogamous species exhibit at least some extra-pair reproduction due to females promiscuously seeking copulations with males that are not their social mates (Griffith et al., 2002). This mating pattern implies that there exists more variation in male reproductive success than would be expected if males and females simply mated within their pair bonds. Also in fishes, males can clandestinely participate in the spawning events of other males that are already paired with a female (or females). If such cuckoldry is performed by bachelor males that do not, and will not, pair with females, then cuckoldry can reduce the variance in reproductive success among all males in the population (Jones et al., 2001; Candolin & Vlieger, 2013; Bose et al., 2018). If, however, cuckoldry is performed by paired males, or if males switch between pairing and cuckolding during their lifetime, then the effects of cuckoldry on variance in reproductive success are more difficult to predict (Collet et al., 2012; Isvaran & Sankaran, 2017; Raj Pant et al., 2022).

The mating patterns of cichlid fishes have attracted strong research interest in the context of behavioural and evolutionary studies, and in a number of species, both their social and genetic mating systems have been described. Some socially monogamous cichlids have been found to be genetically monogamous (Taylor et al., 2003; Egger et al., 2006; Takahashi et al., 2012; Schaedelin et al., 2015), whereas others express differing rates of extra-pair paternity (Lee-Jenkins et al., 2015; Bose et al., 2018). Polyandrous spawning occurs to variable degrees in those maternal mouthbrooders where females do not form pair bonds with their mates (Kellogg et al., 1995, 1998; Parker & Kornfield, 1996; Sefc et al., 2009, 2012; Anderson et al., 2016), and in group-living cichlids, the degree to which reproduction is monopolized by dominant individuals or is obtained by subordinates or neighbouring group members varies among species (Awata et al., 2005; Dierkes et al., 2008; Stiver et al., 2009; Bose et al., 2022).

Cichlids display a high diversity of mating behaviours, which can also vary spatially and temporally both within and across species. In shell-breeding lamprologine cichlids, degrees of polygyny range from social monogamy to large harems, and this variation occurs both within and among populations of the same species (reviewed in Sefc, 2011). Studies on both pair bonding and non-pair bonding cichlids have revealed that males of some species experience high levels of paternity losses within their broods and this can be accompanied by considerable variance in multiple paternity rates across broods (Sefc et al., 2009; Anderson et al., 2016; Zimmermann et al., 2019). In two species where brood parentage has been assessed on several occasions from the same population, multiple paternity has varied across time points; in particular, broods sampled after the rainy season have shown higher rates of multiple paternity than broods collected towards the end of the dry season (Sefc et al., 2008, 2009; Bose et al., 2018). Both species—*Ctenochromis* (now: *Shuja*) *horei* (Günther, 1894), a maternal mouthbrooder and *Variabilichromis moorii* (Boulenger, 1898), a substrate breeder—inhabit the shallow rocky littoral of Lake Tanganyika and seasonal variation in environmental factors may be a driver of this temporal variation in brood paternity (Sefc et al., 2009). In fish, potential drivers of seasonal plastic variation in reproductive strategies include food availability, population density, sex ratios, predation pressure and habitat characteristics (Nakano & Nagoshi, 1990; Rossiter, 1995; Rossiter & Yamagishi, 1997; Matsumoto & Kohda, 1998; Magee & Neff, 2006; Monroe et al., 2016).

In this field study, we compiled a time series dataset of brood parentage that we collected from the same population of *Variabilichromis moorii*, a socially monogamous cichlid, across a 3-year period comprising five field excursions. We examined whether variation in the genetic parentage of broods follows a seasonal pattern, testing for recurrent differences between broods spawned during the rainy season and the dry season. Cuckoldry in *V. moorii* is performed by smaller, unpaired, non-territorial males and can lead to substantial paternity losses for paired males (Bose et al., 2018), which in turn affects the payoffs that the cuckolded males receive from brood care (Zimmermann et al., 2019). For females, cuckoldry may also interfere with their preferences regarding who should sire their offspring. Since mate preferences can vary temporally in response to changing environmental conditions (Milner et al., 2010; Moura & Gonzaga, 2017; Frommen et al., 2022), we also investigated whether mate choice in *V. moorii* varied across seasons or years. As in other socially monogamous cichlids (*Eretmodus cyanostictus* (Boulenger 1898): Morley & Balshine, 2003; *Amatitlania nigrofasciatum* (Günther, 1867): Wisenden, 1995; *Neolamprologus caudopunctatus* (Poll, 1978): Schaedelin et al., 2015; *Pelvicachromis taeniatus* (Boulenger, 1901): Baldauf et al., 2009), *V. moorii* show positive size-assortative pairing (Karino, 1997; Zimmermann et al., 2019). Temporal covariation between rates of multiple paternity and the strength of size-assortative pairing would suggest that both processes are influenced by the same environmental factors, or that they influence each other, e.g., when females respond to expectations of cuckoldry by less stringent mate choice.

## Material and methods

### Sample collection and microsatellite genotyping

*V. moorii* is a lamprologine cichlid found in shallow littoral waters along the rocky shores of Lake Tanganyika, where territories of solitary adults and social pairs can be densely clustered (Sturmbauer et al., 2008). Females attach their eggs to the surfaces of rocks and once hatched, the fry hover in the centre of their parents’ territory where they remain until independence (Zimmermann et al., 2021). Brood sizes can reach > 100 fry per territory (Rossiter, 1991; Zimmermann et al., 2019). Breeding occurs continuously across the year and brood care occurs over a period of approximately 100 days (Rossiter, 1991). A breeding pair guards only one cohort of offspring at a time, and neighbouring pairs may be found caring for broods of different ages from one another (Rossiter, 1991). *V. moorii* are conventionally classified as socially monogamous and biparental, but molecular data has revealed frequent cuckoldry (Bose et al., 2018) and behavioural observations suggest that male defence behaviour is more strongly driven by territory retention than by brood protection (Zimmermann et al., 2021).

Over the course of five field seasons, we collected tissue samples from brood-tending parents and their fry, which were used in genetic parentage studies on cuckoldry and brood care (Bose et al., 2018, 2019; Zimmermann et al., 2019, 2021). Here, we used the genetic parentage data from 95 broods out of the above-mentioned studies and extended the data set by adding parentage data of 42 broods sampled specifically for the present study (see Table 1 for sample sizes per field trip). During sampling, we measured the body sizes of the brood tenders (total length, TL, to the nearest 0.1 cm) and determined the water depth at which their territories were located (to the nearest 0.1 m). For each field trip, we returned to the same study quadrat by the eastern shore of Mutondwe Island (~ 100 m × ~ 50 m, depth range: 1.7–12.1 m) in the south of Lake Tanganyika, Zambia (8° 42′ 29.4″ S, 31° 07′ 18.0″ E). The field trips took place in late September to late October 2015 (referred to as field trip ‘2015-dry’), April 2016 (‘2016-rainy’), October to early November 2017 (‘2017-dry’), April 2018 (‘2018-rainy’) and September to early October 2018 (‘2018-dry’). The climate at our study site is characterized by three seasons: cool and dry from May to August, hot and dry from September to November, and warm and wet from December to April. Hence, the broods collected between September and November were spawned in the dry season, and broods collected in April were spawned in the rainy season. See (Bose et al., 2018) for more details on our sample collection methods in the field.

**Table 1:** Summary statistics related to parentage and assortative mating per field trip and season

Trait	October 2015 (dry season)	April 2016 (rainy season)	October 2017 (dry season)	April 2018 (rainy season)	September 2018 (dry season)	Dry seasons pooled	Rainy seasons pooled
Number of broods	33	42	19	21	22	74	63
Brood size (mean ± SD, range)	28.55 ± 20.44, 6–94	34.67 ± 21.27, 9–102	60.63 ± 26.31, 12–119	43.48 ± 23.99, 9–97	41.55 ± 25.65, 11–102	31.59 ± 21.19, 5–95	32.24 ± 17.81, 6–96
Marker polymorphism: sample size *N* and number of loci, mean H_e_	*N* = 130, 14 loci, mean H_e_ = 0.879	*N* = 98, 14 loci, mean H_e_ = 0.876	*N* = 77, 9 loci, mean H_e_ = 0.882	*N* = 89, 9 loci, mean H_e_ = 0.875	*N* = 157, 9 loci, mean H_e_ = 0.876	na	na
Paternity share of brood-tending male (mean ± SD, median, range)	0.73 ± 0.31, 0.82, 0–1	0.46 ± 0.32, 0.46, 0–1	0.58 ± 0.26, 0.57, 0.03–1	0.41 ± 0.23, 0.42, 0–1	0.70 ± 0.35, 0.86, 0.06–1	0.68 ± 0.32, 0.70, 0–1	0.44 ± 0.29, 0.43, 0–1
Sires per brood (mean, median, range)	2.36 ± 1.92, 2, 1–9	3.52 ± 2.02, 3, 1–10	2.95 ± 1.08, 3, 1–5	3.81 ± 1.57, 4, 1–7	2.36 ± 1.53, 3, 1–6	2.51 ± 1.62, 2, 1–9	3.62 ± 1.87, 3, 1–10
Female body size (cm) (mean ± SD)	8.38 ± 0.34	8.16 ± 0.42	8.85 ± 0.50	8.36 ± 0.65	8.81 ± 0.48	8.63 ± 0.48	8.23 ± 0.52
Male body size (cm) (mean ± SD)	8.26 ± 0.41	8.00 ± 0.40	8.54 ± 0.49	7.90 ± 0.66	8.56 ± 0.78	8.43 ± 0.58	7.96 ± 0.50
Body size correlation (Pearson *r*)	*r* = 0.56, *P* = 0.001	*r* = 0.24, *P* = 0.145	*r* = 0.81, *P* < 0.0001	*r* = 0.45, *P* = 0.042	*r* = 0.32, *P* = 0.146	*r* = 0.54, *P* < 0.0001	*r* = 0.34, *P* = 0.009

Parentage exclusion probabilities ranged from 0.9999990683 (April 2018) to 0.9999999998 (September 2015)

*H*_*e*_ expected heterozygosity

DNA extraction and microsatellite genotyping were carried out as described in Bose et al. (2018). Brood tending parents and fry collected in 2015 and 2016 were genotyped at 14 microsatellite loci (Bose et al., 2018), whereas samples collected during the later trips were genotyped at a subset of 9 loci (Zimmermann et al., 2021). Population allele frequencies were estimated from population samples collected during the same field season as the broods. Gene diversity and parentage exclusion probabilities of the microsatellite marker sets are reported in Table 1. Parentage analyses were performed using COLONY (v. 2.0.6.1, Jones & Wang, 2010) as described in Bose et al. (2018). COLONY partitions broods into full sib groups and assigns fry to candidate genotyped parents (here, brood-tending pairs) and to unsampled parents (here, cuckolders). The output of COLONY was manually corrected for over-estimated sire numbers (Sefc and Koblmüller, 2009) as described in Bose et al. (2018). The paternity share of each brood-tending male, and each of their cuckolders, was calculated as the proportion of fry that they had sired out of the total number of fry that were assigned as offspring of the brood-tending male’s female partner. Additional fry that were not related to either brood-tending parent were found in 20–40% of territories across the field trips and amounted to 7–15% of fry in a territory (averages across territories for each field trip). We assumed that the unrelated fry had migrated into the territory (Satoh et al., 2021), and that only those fry assigned to the paired, brood-tending female were spawned on the territory.

### Statistical analysis

Statistical analyses were conducted in R v. 4.1.3 (R Core Team, 2022). We fit generalized linear mixed models (GLMMs; R package glmmTMB, Brooks et al., 2017) to test for temporal differences in paternity shares and sire numbers per brood, and we tested for pairwise differences among the five field trips. We also fit analogous GLMMs with the data grouped by season and tested for overall differences between the rainy and dry season. In each GLMM, we initially included water depth (continuous variable), average body size of the two parents (continuous variable), and either field trip or season (categorical variables) as predictors. We also included an interaction between the body size of the parents and field trip or season. The significance of the interaction terms was tested with likelihood ratio tests (LRT). The effect of depth as well as the LRT testing the interaction between body size of the parents and field trip/season were not significant and these terms were, therefore, omitted from the final models. Thus, the final models included the average body size of the two parents and either the field trip (5-level categorical variable) or the season (2-level categorical variable) as predictor variables along with an observation-level random intercept to account for overdispersion (Harrison, 2014). Checks for collinearity of predictor variables were run for all models with more than one predictor using the function check.collinearity from package ‘performance’ (Lüdecke et al., 2021) and all VIF values were found to be < 1.5. Pairwise contrasts between field trips were performed with the ‘multcomp’ R package (Hothorn et al., 2008) using the Tukey method.

GLMMs examining temporal variation in paternity shares of brood-tending males and cuckolders were fit with binomial error distributions. Here, paternity share of the brood-tending male was the response variable and was specified by a matrix of two columns, one representing the number of fry sired by the brood-tending male and the other representing the number of fry sired by his cuckolders. GLMMs examining temporal variation in the paternity shares of individual cuckolders were also fit with binomial error distributions. Here, the paternity share of each cuckolder was the response variable and was specified by a matrix of two columns, one representing the number of fry sired by the cuckolder and the other the number of remaining offspring that had been spawned by the brood-tending female. Since broods could have had offspring from more than one cuckolder, ‘territory ID’ was included as additional random intercept in these models.

To test for a correlation between a brood’s sire number and the paternity share of the brood-tending male, we fit a GLMM with a binomial error distribution. Paternity share of the brood-tending male was fit as the response variable, with number of sires as a predictor variable, and ‘field trip’ as a random intercept.

GLMMs examining temporal variation in the number of sires per brood were fit with Poisson-distributed errors. We included average body size of the parents and either field trip or season as predictor variables. As above, an observation-level random intercept was included to account for overdispersion.

Finally, we used linear models to test for temporal variation in the strength of size-assortative pairing. Male size was included as a continuous response variable, and we included female size (continuous variable), field trip or season, and their interaction term as predictor variables. We also calculated Pearson correlation coefficients between the male and female partner body sizes for each field trip and each season. We used a paired t-test to examine size differences between the sexes within social pairs, and a Welch’s two-sample t-test to examine body size dimorphism in the population.

## Results

### Paternity shares of brood-tending males are higher in the dry season

**Fig. 1:**
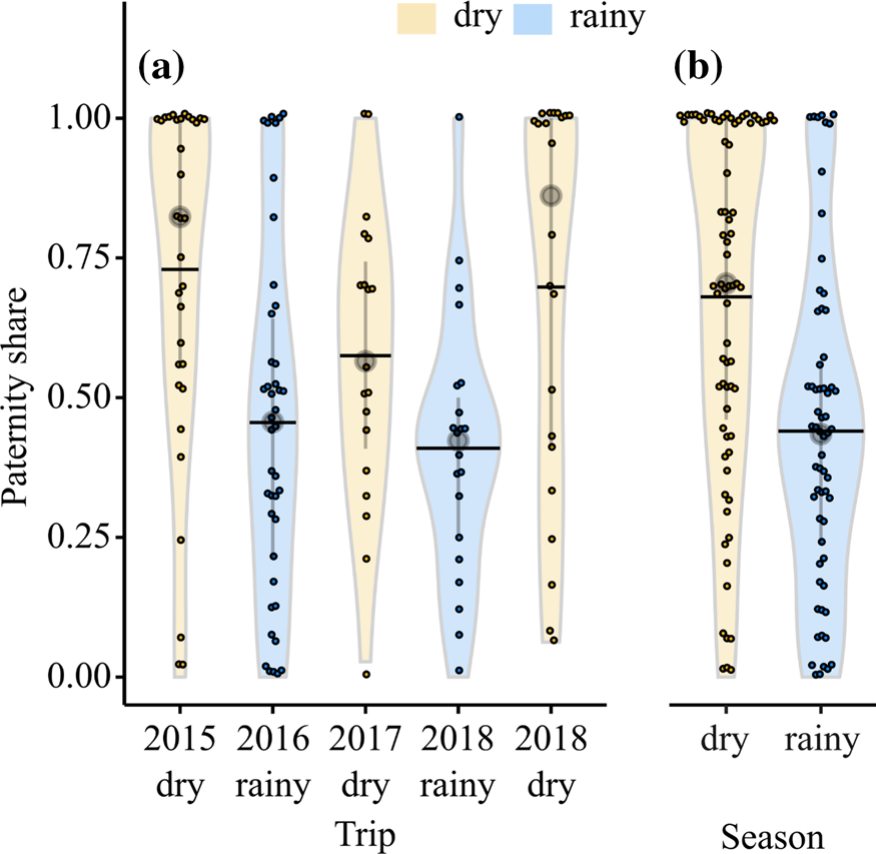
Paternity shares of brood-tending males per field trip (**a**) and per season (**b**). Coloured dots represent the paternity shares of individual males with different colours representing different seasons (yellow = dry season, blue = rainy season) and individual dots were allowed to jitter by a share of 0.01 to avoid overlap. The violin plots depict the distributions of paternity share values per brood, including sample means (horizontal lines), sample medians (grey circles) and interquartile ranges (grey vertical lines)

Paternity shares of brood-tending males varied among field trips (LRT: $\chi^{2}$ = 23.2, df = 4, *P* = 0.0001) and were higher in the dry seasons than in the rainy seasons (Fig. 1a, b). The pairwise contrasts between the dry seasons and the rainy seasons were statistically significant except for contrasts involving the 2017-dry season (October) sample (where the contrasts were not significant, but in a consistent direction). None of the same-season contrasts were significant (Table 2). There was a significantly negative effect of the pair’s average body size on paternity share of the brood-tending male (GLMM, est. = −1.02, *z* = −2.04, *P* = 0.041). Pooling data from multiple field trips by season yielded consistent results with a significant negative effect of body size (GLMM, est. = −1.01, *z* = −2.11, *P* = 0.035) and significantly higher paternity shares of the brood-tending males in the dry season compared to the rainy season (GLMM, est. = 2.16, *z* = 4.47, *P* < 0.0001).

**Table 2:** Output of linear mixed effects model examining paternity share of brood-tending males

	Estimate	SE	*z* value	*P *value
Between seasons contrasts				
2015-dry – 2016-rainy	2.17	0.61	3.54	**0.004**
2015-dry – 2018-rainy	2.18	0.69	3.15	**0.014**
2017-dry – 2016-rainy	1.44	0.72	2.01	0.26
2017-dry – 2018-rainy	1.46	0.78	1.86	0.34
2018-dry – 2016-rainy	2.84	0.74	3.82	**0.001**
2018-dry – 2018-rainy	2.86	0.81	3.55	**0.003**
Dry season contrasts				
2017-dry – 2015-dry	−0.72	0.72	−1.01	0.85
2018-dry – 2015-dry	0.68	0.73	0.93	0.89
2018-dry – 2017-dry	1.40	0.76	1.84	0.35
Rainy season contrast				
2018-rainy – 2016-rainy	−0.015	0.64	−0.024	1.00

Pairwise comparisons were made using the Tukey method in the “multcomp” R package. Significant values are in bold

### Sire number per brood is higher in the rainy season

**Fig. 2:**
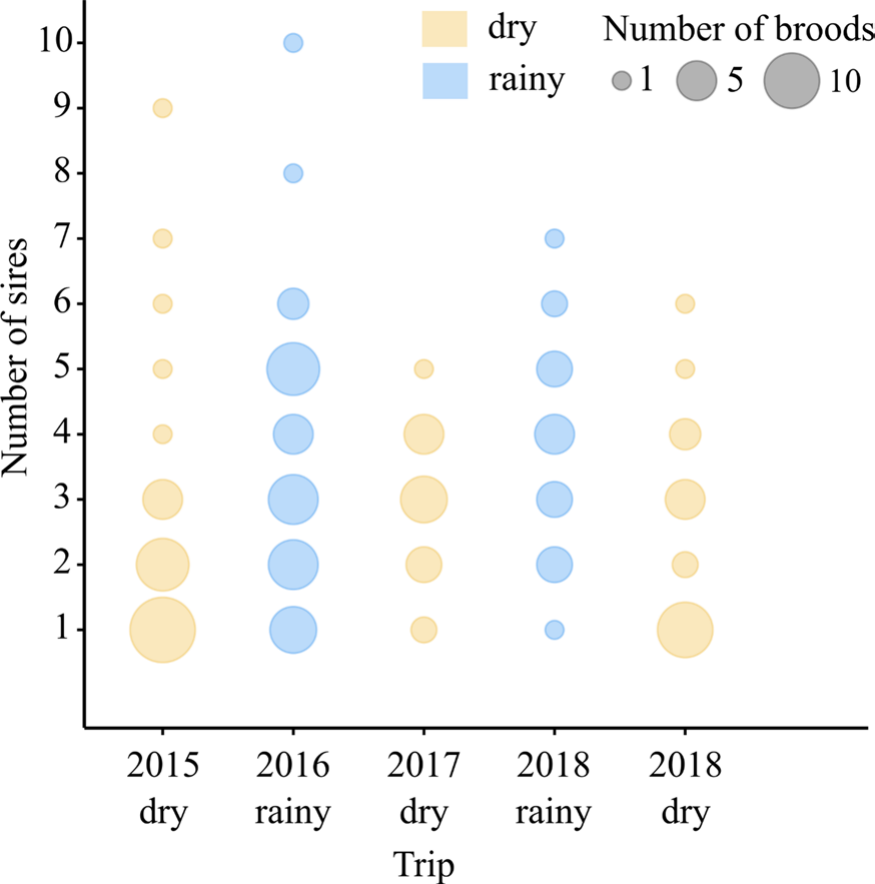
Sire numbers per brood, broken down by field trip. The sizes of the circles represent the number of broods with a given number of sires

The paternity shares of brood-tending males were negatively correlated with the number of additional sires that fertilized eggs in their female partners’ broods (GLMM, est. = −0.56, *z* = −24.77, *P* < 0.0001). Sire numbers per brood varied among field trips (LRT, $\chi^{2}$ = 22.9, df = 4, *P* = 0.0001; Fig. 2) and were positively correlated with the pairs’ mean body sizes (GLMM, est. = 0.38, *z* = 3.39, *P* = 0.0007). Pairwise comparisons between field trips indicated fewer sires per brood in dry compared to rainy seasons (*P* values < 0.01), whereas none of the same-season comparisons revealed significant differences in sire numbers (Table 3). Pooled by season, the average number of sires per brood was positively correlated with the brood-tending pair's body size (GLMM, est. = 0.36, *z* = 3.38, *P* = 0.0007) and was higher in the rainy season compared to the dry season (GLMM, est. = 0.51, *z* = 4.63, *P* < 0.0001).

**Table 3:** Output of linear mixed effects model examining sire number per brood

	Estimate	SE	*z* value	*P *value
Between seasons contrasts				
2015-dry – 2016-rainy	− 0.46	0.15	− 3.12	**0.015**
2015-dry – 2018-rainy	− 0.48	0.16	− 2.96	**0.025**
2017-dry – 2016-rainy	− 0.44	0.18	− 2.52	0.085
2017-dry – 2018-rainy	− 0.46	0.18	− 2.51	0.087
2018-dry – 2016-rainy	− 0.66	0.18	− 3.70	**0.002**
2018-dry – 2018-rainy	− 0.68	0.19	− 3.64	**0.003**
Dry season contrasts				
2017-dry – 2015-dry	0.017	0.18	0.092	1.00
2018-dry – 2015-dry	− 0.20	0.19	− 1.09	0.81
2018-dry – 2017-dry	− 0.22	0.19	− 1.15	0.78
Rainy season contrast				
2018-rainy – 2016-rainy	0.019	0.14	0.14	1.00

Pairwise comparisons were made using the Tukey method in the “multcomp” R package. Significant values are in bold

### Higher average paternity shares for brood-tending than for cuckolder males in all seasons

**Fig. 3:**
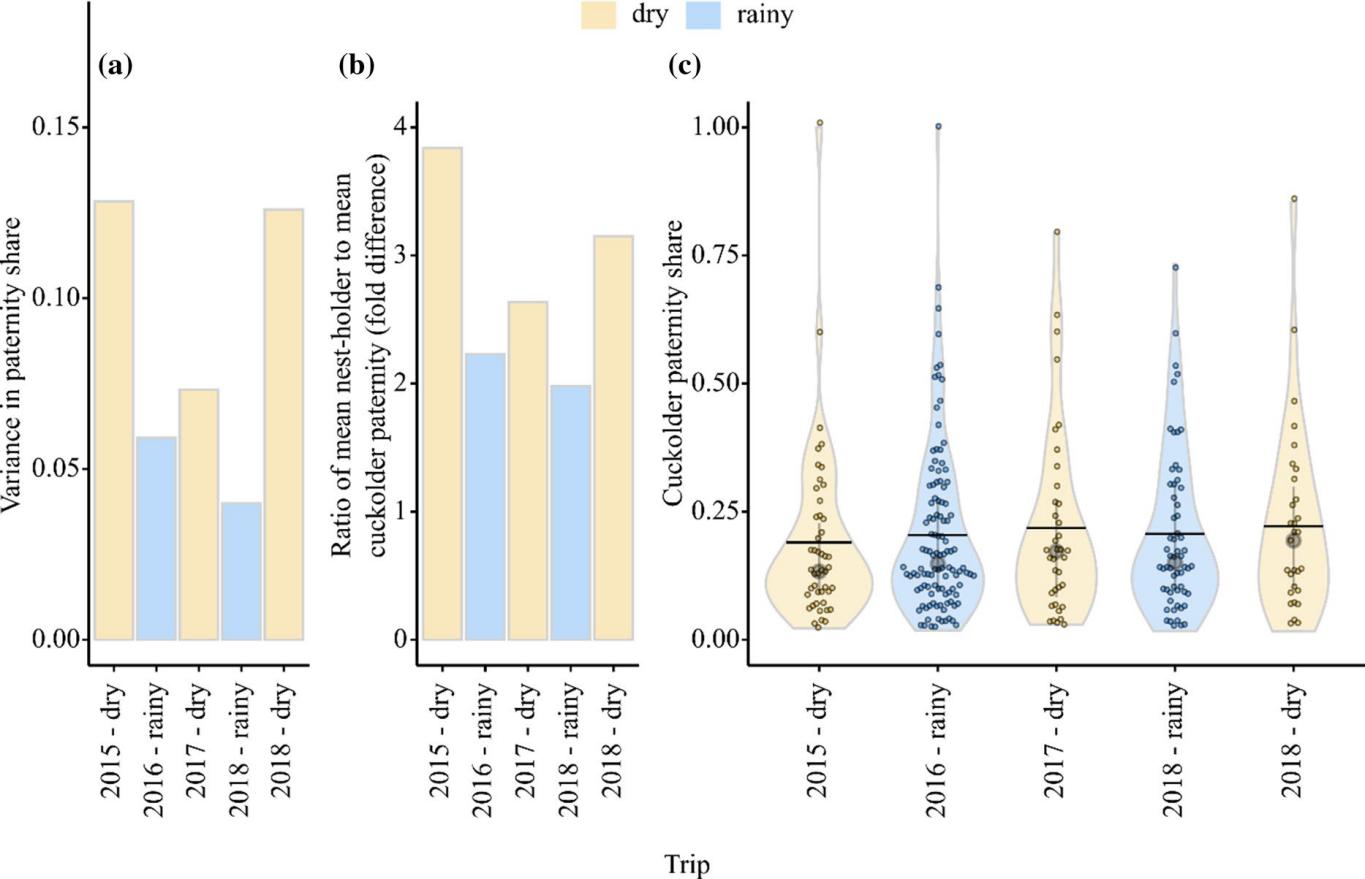
Ratio of average paternity shares of brood-tending males relative to cuckolder males, broken down by field trip (**a**). Paternity shares of individual cuckolders, broken down by field trip (**b**). Coloured dots represent the paternity shares of individual cuckolders with different colours representing different seasons (yellow = dry season, blue = rainy season) and individual dots were allowed to jitter by a share of 0.01 to avoid overlap. **c** The violin plots depict the distributions of paternity share values per brood, sample means (horizontal lines) and sample medians (grey circles) with interquartile ranges (grey vertical lines)

Average paternity shares of brood-tending males were 2.0 to 3.8 times higher than average paternity shares of cuckolders, and this bias was always stronger in the three dry seasons compared to the two rainy seasons (Fig. 3a). The paternity shares of individual cuckolders did not vary with the mean body size of the brood-tending pair (GLMM, est. = −2.51, *z* = −1.57, *P* = 0.12) nor did they differ among field trips (LRT, $\chi^{2}$ = 2.90, df = 4, *P* = 0.58; Fig. 3b; *n* = 93 nests with cuckolder paternity).

### Positive size-assortative pairing does not differ significantly between seasons

**Fig. 4:**
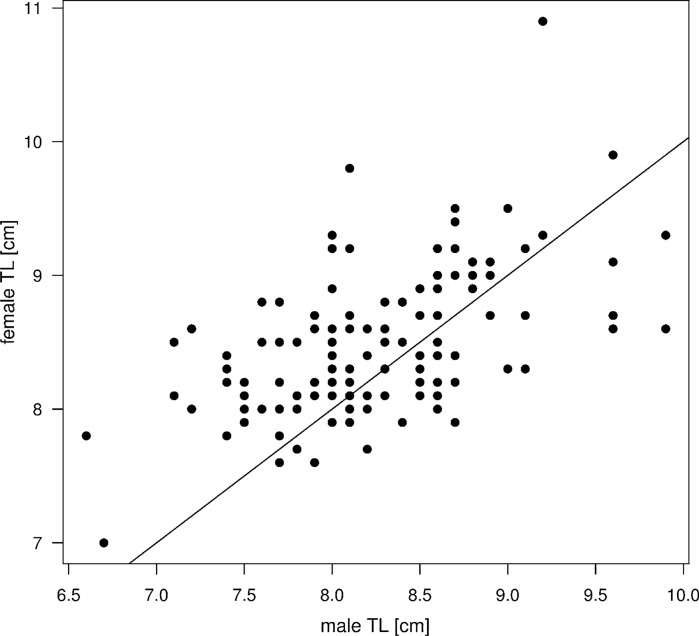
Scatterplot of female versus male body size (total length). The solid crossline corresponds to a 1:1 fit

When pooling across all five field trips, there was a significant correlation between the body sizes of male and female brood-tending partners (Pearson *r* = 0.53; *t* = 7.1, df = 128, *P* < 0.0001; Fig. 4; see Table 1 for results per field trip and per season), and the strength of this correlation did not differ significantly among field trips or between the dry and rainy seasons (LRT; for interaction with field trip: *F* = 0.91, df = 4, *P* = 0.46; for interaction with season: *F* = 3.15, df = 1, *P* = 0.08). Across samples, females were on average 0.2 cm larger than their male partners (paired *t-*test, *t* = 4.92, df = 129, *P* < 0.0001), and this difference corresponds to the population-level size difference of 0.2 cm between males and females (Welch’s two-sample *t-*test, *t* = 3.47, df = 263.04, *P* = 0.0006).

## Discussion

In this study, we tested whether two factors that can influence the reproductive success of brood-tending males exhibited seasonal temporal variation. One factor was cuckoldry pressure by unpaired floater males, and the other factor was mate choice in relation to the body sizes of potential mating partners. We discuss each of these in turn.

The paternity shares of brood-tending males varied among field trips in a seasonal pattern: paternity loss suffered by brood-tending males was higher in the rainy compared to the dry season, and the number of successful cuckolders per brood was also higher in the rainy season. Surprisingly, despite fish mating systems being especially rife with alternative reproductive tactics (Taborsky, 2008), temporal variation in cuckoldry rates has been monitored in only a few fish species to date, and rarely across extended periods of time that span multiple years. For example, a study examining the rate of reproductive success by sneaker males in a Norwegian population of two-spotted gobies, *Gobiusculus flavescens*, (Fabricius, 1779) revealed no significant differences in paternity rates of nest-owning males between early and late spawning season (Monroe et al., 2016). In two species of sunfish, paternity shares of brood-tending males varied between early, mid and late broods of a breeding season, possibly driven by temporal variation in the defence capabilities of nest owners or cuckolder abundance (Neff & Clare, 2008). In the plainfin midshipman fish, *Porichthys notatus* (Girard, 1854), average paternity shares of guarding males increased over the course of a breeding season, ranging from less than 10% to near 100%, a pattern that is likely driven by declining occurrences of nest take-overs as the season progresses (Cogliati et al., 2013). Rates of multiple paternity also differed across time between two samples taken from a sailfin molly population in Florida (Trexler et al., 1997) and from a population of the Lake Tanganyika cichlid *Ctenochromis* (*Shuja*) *horei* (Sefc et al., 2009).

Many cichlids in Lake Tanganyika breed throughout the year, and they can experience environmental variation in response to seasonal (rainy versus dry seasons) weather conditions. For instance, rainfall increases the influx of sediment into the lake, and we have repeatedly encountered poor underwater visibility during field trips early in the year (March, April) and more stable conditions of good visibility between September and November (e.g., personal observations of S. Koblmüller and authors of this study). Interestingly, the dry season ended prematurely in 2017 (Zimmermann pers. obs.), and the difference in paternity shares and sire numbers in pairwise comparisons between *V. moorii* broods sampled in October 2017 and the rainy season samples was less pronounced than was the case for October 2015 and September 2018. Water turbidity could foreseeably affect cuckolders in two opposing ways. In turbid water, potential cuckolders may be able to approach a target nest more closely before being detected and driven away by the nest residents, with the implication that cuckoldry may be more successful when visibility is poor (e.g., Candolin & Vlieger, 2013). On the other hand, reduced visibility may make it more difficult for potential cuckolders to identify spawning opportunities, which would lead to reduced rates of cuckoldry (e.g., Vlieger & Candolin, 2009). Females that are aware of the presence of cuckolders may respond either by interrupting or delaying spawning to avoid having their eggs fertilized by non-preferred males, or they may display a preference for spawning in the presence of sneaking males (Reichard et al., 2007). In either case, the socially bonded male and female’s responses to the presence of cuckolders may depend on their ability to detect these individuals in their environment, and thus, might be influenced by water turbidity, which is worse in the coastal shallow waters of Lake Tanganyika during the rainy seasons. In addition to fluctuations in water turbidity, weather cycles might also induce variation in other factors such as primary productivity, altering food availability that could support higher or lower population densities, and hence, cuckolder pressure.

In agreement with the seasonal signature of multiple paternity presented here, a previous study by Sefc et al. (2008), examining brood paternity in a neighbouring population of *V. moorii*, found very high rates of multiple paternity within their sample that was also taken during a rainy season. Similarly, a population of another maternal mouthbrooder from Lake Tanganyika, *C.* (*S.*) *horei*, experienced higher rates of multiple paternity in the rainy season than in the preceding dry season (Sefc et al., 2009). In *C.* (*S.*) *horei*, females do not form pair bonds with their mates, but may successively spawn with different males and be targeted by sneaking males (Ochi, 1993). Spanning a longer time scale, our current data further corroborate the existence of a seasonal pattern, but dedicated future studies are necessary to address the underlying mechanisms more directly.

In *V. moorii*, although cuckoldry is very prevalent, nearly all cuckoldry is perpetrated by unpaired males from a large pool of non-territorial, floater individuals (Bose et al., 2018). We, therefore, assumed that, in the current study, every full sib group of offspring reconstructed in the genotyped broods represented offspring from a different sire. Hence, we could use the paternity shares of the brood-tending males and of their cuckolders to compare their relative reproductive success. In each of our field trips, the average paternity shares of brood-tending males were at least twofold higher than those of cuckolders. Hence, in each of the five periods covered by our sampling, breeding as a paired territorial male conferred higher reproductive success than cuckoldry, although the difference varied between field trip samples (from twofold to almost fourfold). The average success of individual cuckolders did not vary across seasons, indicating that the higher paternity losses suffered by brood-tending males in the rainy season were due to higher numbers of successful cuckolders and not to greater success of individual cuckolders.

We also examined whether mate choice, as captured by the size-assortative pairing of breeding *V. moorii*, displayed seasonal variation. Mate preferences and choosiness are influenced by numerous abiotic and biotic factors and may co-vary with environmental fluctuations (Jennions & Petrie, 1997; Candolin, 2019). For example, in turbid water, mating success of male sand gobies was less skewed towards large males than under clear conditions (Järvenpää & Lindström, 2004), and in sticklebacks, female preferences for male courtship activity were influenced by the presence of predators (Frommen et al., 2022). *V. moorii* are sexually monomorphic and it is unclear which traits are used in mate assessments and mate choice. Like many other animal species (Jiang et al., 2013), including socially monogamous fish (Wisenden, 1995; Morley & Balshine, 2003; Baldauf et al., 2009; Schaedelin et al., 2015), *V. moorii* engage in positive size-assortative mating. Both male and female fish might benefit from large mates (Lindström & Pampoulie, 2005; Barneche et al., 2018), and pair formation may be influenced by both female and male mate preferences. The strength of the positive correlation between male and female body sizes did not differ among our field trips, suggesting that the mechanisms that led to size-assortative pairings were stable across time.

In this study, we show that even when reproduction occurs continuously throughout the year without a defined breeding season, distributions of reproductive success can vary over time and can show pronounced seasonal signatures. This can be the case when animal mating patterns themselves, or conditions that affect mating patterns, vary with environmental fluctuations (Jennions et al., 2012; e.g., Robinson et al, 2008; Cornwallis & Uller, 2010). Our results, therefore, imply that assessments of mating patterns taken from single snapshots in time can only partially capture the distributions of parentage in a system, which can complicate our ability to make generalizations about species-level mating patterns based on single time-point observations. This highlights the utility of longitudinal datasets in capturing the plasticity of mating patterns and describing the extent to which they vary across time within a species or population.

## Supplementary Information

Below is the link to the electronic supplementary material.

Supplementary file1 (XLSX 39 kb)

## Data Availability

The datasets analyzed during the current study are available in the supplementary information.
